# Newly Developed Topical Cefotaxime Sodium Hydrogels: Antibacterial Activity and* In Vivo* Evaluation

**DOI:** 10.1155/2016/6525163

**Published:** 2016-05-24

**Authors:** Azza S. Zakaria, Samar A. Afifi, Kadria A. Elkhodairy

**Affiliations:** ^1^Department of Pharmaceutics, College of Pharmacy, King Saud University, P.O. Box 22452, Riyadh 11495, Saudi Arabia; ^2^Department of Microbiology, Faculty of Pharmacy, Alexandria University, P.O. Box 21500, Alexandria 21527, Egypt; ^3^Department of Pharmaceutics, National Organization for Drug Control and Research, P.O. Box 35521, Giza 12561, Egypt; ^4^Department of Industrial Pharmacy, Faculty of Pharmacy, Alexandria University, P.O. Box 21500, Alexandria 21527, Egypt

## Abstract

In an attempt to reach better treatment of skin infections, gel formulations containing Cefotaxime (CTX) were prepared. The gel was formulated using Carbopol 934 (C934), Hydroxypropyl Methylcellulose 4000 (HPMC 4000), Carboxymethylcellulose Sodium (Na CMC), Pectin (PEC), Xanthan Gum (XG), or Guar Gum (GG). Thirteen different formulas were prepared and characterized physically in terms of color, syneresis, spreadability, pH, drug content, and rheological properties. Drug-excipients compatibility studies were confirmed by FTIR and then* in vitro* drug release study was conducted.* In vitro* and* in vivo* antibacterial activities of CTX were studied against wound pathogens such as,* Staphylococcus aureus* (*S. aureus*),* Escherichia coli* (*E. coli*), and* Pseudomonas aeruginosa* (*P. aeruginosa*), using either pure drug or Fucidin® cream as control. F13 provides better spreadability compared to F1 (XG) or F11 (HPMC). Moreover, the release of the drug from hydrogel F13 containing C934 was slower and sustained for 8 h. Stability study revealed that, upon storage, there were no significant changes in pH, drug content, and viscosity of the gels. Also, F13 showed the larger inhibition zone and highest antibacterial activity among other formulations. Histological analysis demonstrated that after single treatment with F13 gel formulation, a noticeable reduction in microbial bioburden occurred in case of both Gram positive and Gram negative bacterial isolates.

## 1. Introduction

Several antimicrobial agents are available in the market in different pharmaceutical dosage forms for the treatment of skin and soft tissue infections (SSTIs). These preparations are designed to be administered either orally or parenterally or topically. Topical preparations have several benefits compared with systemic therapy [1, 2]. Initially, the required concentration for antibiotic activity at the skin target site may be more easily achieved after topical dosing. Moreover, topical administration results in much lower undetectable systemic levels [3]. Additionally, it can avoid an unnecessary exposure of gut flora that may exert selection for resistance. Therefore, topical application of antimicrobial agents is considered an important alternative using the stratum cornea as the target organ of various antibiotic drug treatments [1].

Controlled release of antibiotics at site of infection is a new strategy being employed to treat chronic infections [4, 5]. Localized delivery systems, based on biodegradable polymers, are capable of slowing and controlling drug release for a certain period of time, with initial burst effect to circumvent the infection.

Hydrogels, swollen three-dimensional networks of hydrophilic polymers held together by association bonds or cohesive forces, are of special interest in controlled release applications, because of their soft tissue biocompatibility, the ease with which drugs are dispersed in matrix, and the high degree of control achieved by selecting the physical and chemical properties of polymer network. Hydrogels have been investigated extensively for application as carriers in diffusion-controlled release devices [6]. Hydrogels are a common form of topical application. They achieve sustained release by diffusion from a reservoir through microporous membrane into the skin [5].

Cefotaxime (CTX) sodium as an antimicrobial agent is a semisynthetic broad spectrum third-generation cephalosporin against Gram positive and Gram negative bacteria and some pseudomonal species as well as some anaerobic bacteria [7]. It has activity in presence of some beta-lactamases, both penicillinases and cephalosporinases of Gram negative and Gram positive bacteria. Its spectrum of activity includes most strains of bacterial pathogens responsible for septicaemia, respiratory tract infections, urinary tract infections, soft tissue infections, bone and joint infections, obstetric and gynaecological infections, and other various types of infections [8]. Cephalosporins as an antibiotic group is well known to have minimal side effects compared to other antibiotic groups.

To our knowledge, there is neither marketed topical formulation of this essential group nor research study performed to investigate topical cephalosporin preparations. Based on this knowledge, it was of interest to formulate CTX as topical preparation to treat bacterial skin and soft tissue infections more efficiently.

The present study was conducted to formulate topical hydrogel formulations of CTX. Natural polymers such as Pectin (PEC), Xanthan Gum (XG), Guar Gum (GG), synthetic polymer, namely, Carbopol 934 (C934), and semisynthetic polymers such as Hydroxypropyl Methylcellulose (HPMC) and Carboxymethylcellulose Sodium (Na CMC) were used as gelling agents. Effect of propylene glycol (PG) used as a penetration enhancer on the release had been studied. Hydrogels were evaluated for their physical appearance, rheological behavior, spreadability, drug release, and antimicrobial activity. Since different microorganisms can exist on the margins of chronic wounds [9],* in vitro* and* in vivo* antibacterial activity of CTX was studied against commonly isolated wound pathogens such as* Staphylococcus aureus* (*S. aureus*),* Escherichia coli* (*E. coli*), and* Pseudomonas aeruginosa* (*P. aeruginosa*) [10, 11].

## 2. Experimental

### 2.1. Materials

#### 2.1.1. Materials Used for Gel Preparation

Cefotaxime (CTX) sodium and Hydroxypropyl Methylcellulose 4000 (HPMC 4000) were purchased from Sigma, USA. Xanthan Gum was purchased from Ultrafine (India). Guar Gum was purchased from Premcem Gums Ltd. (India). Sodium carboxymethyl cellulose (Na CMC) and Pectin (Pec) were obtained from Shinestu Chemical Co., France. Carbopol 934 (C934) and Triethanolamine (TEA) were purchased from Loba Chemie, Mumbai, India. Propylene glycol (PG) and sodium benzoate are obtained from Merck, Germany. Fucidin is a product of LEO Pharmaceutical products, Denmark.

#### 2.1.2. Materials Used for Microbiological Studies

*(1) Bacterial Isolates*. A total of six bacterial isolates were used, one standard* E. coli* (*E. coli*) (ATCC 25922) and one* E. coli* clinical isolate and one standard* Pseudomonas aeruginosa* (*P. aeruginosa*) (ATCC 27853) and one clinical isolate. Finally, one standard* Staphylococcus aureus *(*S. aureus*) (ATCC 29213) and one methicillin resistant* S. aureus* clinical isolate were studied in this research. The strains were collected from wound specimens from outpatient departments of King Khalid Hospital, Riyadh, Saudi Arabia.

*(2) Media and Culture Conditions*. All clinical samples were first inoculated onto Sheep Blood Agar (SPML Co. Ltd,, Riyadh, Saudi Arabia), The plates were incubated at 37°C for 24–48 h. Identification of isolates was done according to standard methods described elsewhere [12, 13] and Clinical Laboratory Standards Institute [14]. Isolates were stored in brain heart infusion broth containing 16% (w/v) glycerol at −80°C until further use.

*(3) Growth on Mannitol Salt Agar*. Staphylococcal isolates were reinoculated onto Mannitol Salt Agar (Oxoid, Hampshire, UK) and then the plates were incubated at 37°C for 24–48 h. Mannitol fermentation was observed and recorded.

*(4) Growth on MacConkey and Cetrimide Agar.* Gram negative isolates were reinoculated onto MacConkey Agar (Oxoid, Hampshire, UK) and then the plates were incubated at 37°C for 24–48 h. Lactose fermentation was observed and recorded.* P. aeruginosa* strains were further reinoculated on cetrimide agar (Oxoid, Hampshire, UK) at 37°C for 24 h.

### 2.2. Methods

#### 2.2.1. Fourier Transmitted Infrared Spectroscopy (FTIR)

FTIR spectra were performed to find out any possible drug-polymer interactions. Samples of CTX and of each polymer used (XG, GG, PEC, HPMC, Na CMC, and C934) as well as samples of their physical mixtures in the ratio of 1 : 1 were grounded separately and mixed thoroughly with potassium bromide. A total of 1 mg of each sample and 100 mg of potassium bromide (KBr) were ground uniformly and compressed to form a KBr film disc. Potassium bromide discs were prepared by compressing the powders at a pressure of 5 tones for 5 min in a hydraulic press. Data were scanned over a spectral region from 400 to 4000 cm^−1^ and the obtained spectra were analyzed [15].

#### 2.2.2. Preparation of Cefotaxime (CTX) Sodium Gel Formulations

Drug-loaded hydrogels were prepared according to previously demonstrated procedure [6, 16]. In general, there are four different classes of excipients usually incorporated in order to prepare topical gels which are gelling agents, humectants, preservatives, and vehicles [17]. Different concentrations of various polymers (gelling agents) were postulated. Natural polymers such as GG (in range of 2–5% w/w), XG (in range of 2-3% w/w), PEC (in range of 6-7% w/w), semisynthetic polymers HPMC 4000 (in range of 3-4% w/w), and Na CMC (in 4 and 7% w/w) as well as the synthetic polymers C934 in concentration of 2% w/w were used. Drug-loaded hydrogels were formulated by dispersing the gelling agents slowly in an aqueous-based solution containing CTX (1% w/w), PG (5% w/w, as a humectant), and sodium benzoate (0.25% w/w, as a preservative), with the help of an overhead mechanical stirrer at a moderate speed. pH of C934 hydrogel was adjusted using TEA with stirring until desired pH value was approximately reached (6.8–7). The prepared hydrogels were packed in wide mouth jar covered with screw capped plastic lid and were kept in dark and cool place [18]. Samples were allowed to equilibrate for at least 24 h at room temperature prior to performing rheological measurement [3, 19]. The composition of different prepared CTX hydrogel formulations is given in Table 1.

#### 2.2.3. Physicochemical Evaluation of the Prepared Hydrogels

*(1) Visual Examination*. Cefotaxime hydrogels were inspected visually for their color, homogeneity (appearance and presence of any aggregates), grittiness (presence of particles or grits), and syneresis (phase separation).

*(2) pH Determination*. The pH of various CTX gel formulations was determined using pH meter (Mettler Toledo, Switzerland), which was calibrated before each use with standard buffer solutions. A quantity of 1 g of CTX gel was dissolved in 100 mL freshly prepared distilled water and stored for two hours. The electrode was inserted into the sample solution 10 min prior to recording the reading at room temperature. Each measurement was carried out in triplicate and average pH was calculated [3, 20]. Results are shown in Table 2.

*(3) Spreadability Test*. Spreadability (g·cm/sec) is expressed in terms of time taken in seconds by two slides to slip off from the gel placed between them, under certain load [21]. The standardized weight tied on the upper plate was 20 g and length of the glass slide was 7.5 cm [22]. Spreadability was calculated by using the following formula:

(1) $$\begin{array}{c} Spreadability=\frac{\left(Weight\times Length\right)}{Time}. \end{array}$$

According to the above equation, it is clear that the lesser the time taken for separation of the two slides, the better its spreadability.

*(4) Drug Content Determination*. A specific quantity (100 mg) of each prepared hydrogel was taken and dissolved in 100 mL of phosphate buffer (pH 6.8). The volumetric flask containing the gel solution was shaken for 2 h on mechanical shaking water bath in order to complete drug solubility. This solution was filtered and estimated spectrophotometrically at 254 nm [7] using phosphate buffer (pH 6.8) as blank [23]. This test was performed 3 times and mean value ± SD was calculated.

*(5) Determination of Viscosity*. Viscosity of CTX topical hydrogels was determined using Rheometer (Brookfield R/S, USA) with spindle # C 50-1 having a speed of 50 rpm. All measurements were done in triplicate at room temperature [21].

*(6) In Vitro Drug Release Study*.* In vitro drug* release study was done as mentioned before with some modifications [16]. Drug release from the prepared topical hydrogels was determined using dialysis tubing (MWCO of 12400 D; 99.99% retention, Sigma-Aldrich, USA) placed in the release medium under constant stirring using dissolution apparatus USP II (Erweka DT600, Germany). A quantity of 5 g of each hydrogel formulations were individually packed into dialysis tube with the ends being tightly fastened. Release medium was 500 mL phosphate buffer (pH 6.8). The medium was maintained at 37°C ± 0.5 and stirred continuously at 50 rpm. Aliquots of 5 mL of the release medium were withdrawn at predetermined time intervals (15, 30, 45, 60, 90, 120, 180, 240, 300, 360, and 480 min) over a period of 8 h and replaced by fresh phosphate buffer to provide sink condition. Each withdrawn sample was further diluted with phosphate buffer (pH 6.8) and its absorbance was measured using UV-visible spectrophotometer (Biochrom Libra S22, UK) at a *λ*_max_ of 254 nm. Absorbance was converted to drug concentration using a linear calibration curve and then the cumulative percentage of CTX released was calculated with the help of dilution factor. All measurements were performed in triplicate (*n* = 3).

*(7) Drug Release Kinetic Study*. The data obtained from the* in vitro* release study were analyzed using various kinetic models to describe the mechanism of drug release from the hydrogels. Three kinetic models including zero-order (2), first-order (3), and Higuchi square root models (4) were applied on the release data [6]:

(2) $$\begin{array}{c} Q=K_{0}t, \end{array}$$

where *Q* is the amount of drug release at time *t*, *K*_0_ is zero-order rate constant expressed in units of concentration/time, and *t* is the time. Consider

(3) $$\begin{array}{c} \log Q=\log Q_{0}-\frac{K_{1}t}{2.303}, \end{array}$$

where *C*_0_ is the initial concentration of drug and *K*_1_ is first-order constant. Consider

(4) $$\begin{array}{c} Q=K_{H}t^{1/2}, \end{array}$$

where *K*_*H*_ is the constant reflecting the design variables of the system.

To find out the mechanism of drug release, the release data was fitted in Korsmeyer-Peppas model as follows:

(5) $$\begin{array}{c} \frac{M_{t}}{M_{\infty }}={\text{Kt}}^{\text{n}}, \end{array}$$

where *M*_*t*_/*M*_*∞*_ is fraction of drug released at time *t*, *K* is the release rate constant incorporating structural and geometric characteristics of the tablet, and *n* is the release exponent. The *n* value (diffusion exponent) is used to characterize different release mechanisms [24]. The results are shown in Table 3.

*(8) Stability Study*. Hydrogels F1, F11, and F13 which showed a promising sustained drug release were packaged in air tight plastic container and subjected to stability study. They were stored at room temperature (25°C) and in the refrigerator (4°C) over a period of three months [23, 24]. Physical evaluation of the samples was carried out by visual inspection for phase separation and change in color and odor. Rheological properties were also examined. Furthermore, chemical stability was evaluated by spectrophotometric analysis of drug content and pH measurement [25].

#### 2.2.4. Determination of Minimum Inhibitory Concentration (MIC)

MICs of CTX and the different gel formulations were performed in cation-adjusted Müller Hinton broth (Oxoid, Hampshire, UK) (MHB) by means of microdilution broth method in accordance to National Committee for Clinical laboratory Standards [26] and CLSI documents [27]. Stock solutions of CTX pure drug or each of the formulations under test was prepared in sterile distilled water to reach an initial concentration of 2 mg/mL. Preparation of inocula for broth microdilution testing was performed in accordance with CLSI standard procedures. Briefly, 0.5 McFarland equivalent inoculum of each strain was prepared in normal saline from 18–24 h agar plate culture. The suspension was further diluted to achieve desired inoculums concentration of 10^5^ CFU/mL. 100 *μ*L of aliquot of each strain was then added to a 96-well microtiter plate containing gradient concentrations of either CTX pure drug or the gel formulations under test diluted in double strength MHB. The plates were then incubated at 37°C for 24 h. The turbidity of each well was measured at 490 nm with a microplate ELISA reader. The MIC was defined as the lowest concentration of the antibiotic or the hydrogel that prevented bacterial growth.

#### 2.2.5. Determination of the Isolates Sensitivity

Sensitivity of the standard strains and the clinical isolates under test to various CTX formulations was determined by Kirby-Bauer well diffusion susceptibility test described in CLSI [28]. The exponential phase cultures of the bacterial isolates under test were made in sterile normal saline and adjusted to 0.5 McFarland's standard approximately corresponding to 1-2 × 10^8^ CFU/mL. The cultures were then swabbed on Muller Hinton Agar (MHA) plates (Oxoid, UK) uniformly by means of sterile swab. Equidistance wells were cut in the plates with help of 8 mm borer. In each of these wells the gel solutions and pure drug were placed and the plates were left at ambient temperature for 30 min to allow prediffusion prior to incubation at 37°C for 24 h. Antibacterial activity was estimated by measuring the diameter of inhibition zones. Well diffusion tests were performed in triplicates and antibacterial activity was expressed as the mean diameter of inhibition zone ± standard deviation.

#### 2.2.6. *In Vitro* Wound Model for Assessing Effectiveness of Antibiotic Gel

Susceptibility of the bacterial strains in planktonic form was tested against different gel formulations using the modified quantitative method originally introduced by Hammond et al. [29, 30]. Each hydrogel was challenged against one of the strains tested. The concentration of CTX was 1% in each formulation tested. Three sterile 6 mm cellulose disks were placed on a petri dish with Luria Bertani agar (LB agar) (Oxoid, Hampshire, UK) and 10 *μ*L bacterial suspension containing 10^2^–10^3^ CFU was dropped on each disk. The surface of the plate was covered with a sterile gauze square (5 × 5 cm) saturated with gel weight equivalent to 1% CTX. A sterile gauze square without topical antimicrobial gel was used as a control. A sterile small glass petri dish was placed on the gauze to maintain direct contact between the agent and the inoculated disks. The plates were incubated at 37°C for 24 h, after which the gauze squares were removed and each disk transferred into a sterile tube containing 1 mL phosphate buffered saline (PBS). The tubes were vigorously vortexed three times for 2 min to detach bacteria from the disks. Suspended cells were serially diluted four times (a 10-fold dilution was used) in PBS and 10-*μ*L aliquots of each dilution were inoculated on LB agar plates. These plates were incubated at 37°C for 16 h and the numbers of CFU were counted.

The final result recorded represented 3 × 10^8^ CFU/disk if the bacterial growth was so massive that the number of CFU obtained was uncountable even in the highest dilution.

#### 2.2.7. Topical Antimicrobial Efficacy of C934-CTX Hydrogel Using a Mouse Model 

To evaluate the pharmaceutical potential of C934-CTX hydrogel (F13) as a novel local treatment for SSTIs, antimicrobial effect of this hydrogel was investigated in a murine surgical site infection model.

*(1) Experimental Animals*. The animals used for* in vivo* experiments were 200 g specific-pathogen-free male Wistar albino rats. All animals were obtained from Experimental Animal Care Center, College of Pharmacy, King Saud University (KSU), Riyadh, Saudi Arabia. Rats were housed in stainless steel cages (5 animals/cage). Animals were acclimated with free access to tap water and standard pellet diet (Purina Chow) in a facility with controlled temperature (22–24°C) and humidity (50–60%), on a 12 h light/12 h dark cycle, for at least 1week before the experiments. The protocol of this study was carried out according to the regulations and recommendation of the Animal Research Ethics Committee of College of Pharmacy, KSU, Saudi Arabia.

*(2) In Vivo Wound Model*. In this model, two bacterial clinical isolates were chosen based on being the most common pathogen in SSTIs [31, 32]. These strains were* P. aeruginosa* isolate and MRSA isolate. The Bacterial isolates were initially incubated in trypticase soy agar with 5% glucose for 24 h at 37°C and re-suspended in saline adjusted to 0.5 McFarland turbidity.

Rats of each group were intraperitoneally anesthetized with xylazine (8 mg/kg body weight) and ketamine (30 mg/kg body weight), the hair on the lower back was shaved and the skin was cleansed with antiseptic solution (10% povidone-iodine solution). A 1-cm-long, full thickness incision wound was created on the dorsal side of the rat. Approximately 1 cm of silk suture infected with either* P. aeruginosa* isolate or MRSA isolate (approximately 5 × 10^3^ cells/cm of suture) was placed into the wound and secured in the skin by knotting. One single suture was attached over the middle of the incision. The animals were returned to individual cages [33].

*(3) Experimental Protocol*. Rats were randomly allocated into six groups. For each bacterial strain, three challenged groups received the following; Group I (GI); Negative control group (challenged with the bacteria and did not receive any antibiotic). Group II (GII): Positive control group (challenged with the bacteria and treated with a Fucidin cream). This cream was used as an available marketed topical antibiotic formulation containing 2% fusidic acid for the sake of comparison with the formulated hydrogel and group III (GIII): Challenged group with the bacteria and treated with C934-CTX hydrogel 1% (F13).

Antibiotic treatments were started 24 h after incision and suturing. After 24 h, the animals were sacrificed and 1 cm^2^ tissue was cut from the wound area in order to examine the effectiveness of the formulated gel F13 for infection treatment. Equal sections of the isolated skin tissues were used for reading bacterial bioburden. Viable counts of bacteria per section were analyzed in tissue homogenate.

Quantification of viable bacteria in the homogenate was done by culturing serial 10-fold dilutions of the bacterial suspension onto nutrient agar plates. The plates were incubated at 37°C for 24 h and the organisms were quantified by counting the number of CFU/section.

Other tissue sections were further used for histological study. They were fixed with formalin and embedded in paraffin. Consequently, the sections were stained using Hematoxylin and Eosin (H&E), and then they were dried overnight. The stained tissues were microscopically examined and the most detailed and clear tissue slides were chosen [34].

## 3. Results and Discussion

### 3.1. Fourier Transmitted Infrared Spectroscopy (FTIR)

**Figure 1:**
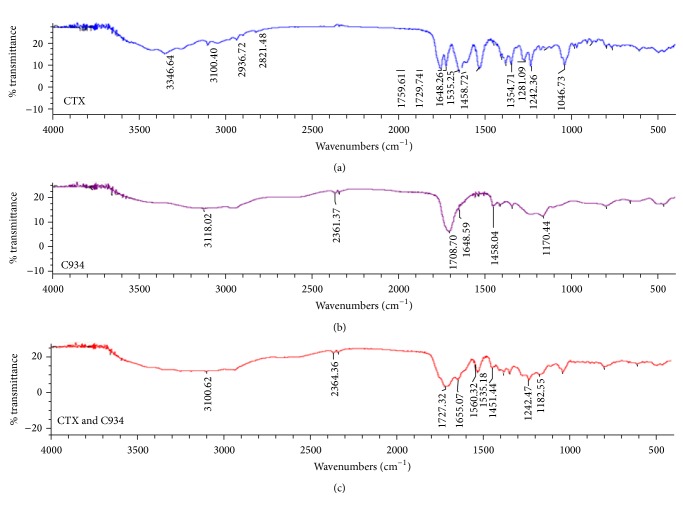
FTIR spectra of CTX (a), C934 (b), and physical mixture of CTX and C934 (c).

FTIR spectrum of free CTX (Figure 1(a)) showed that the characteristics peaks are at 3430 and 3347 cm^−1^ due to NH stretching (NH2) and the peaks at 2938, 1760, 1729, and 1647 cm^−1^ are attributed to aliphatic C–H stretching, C=O (*β*-lactam) stretching, C=O (carboxylic ester), and C=O (amide) stretching, respectively [35]. In addition, the spectrum showed other characteristic peaks at 1610 and 1536 cm^−1^ which are corresponded to C=C and C=N stretching, respectively. Other peaks appear at 1386 and 1355 cm^−1^ due to aliphatic C–H bond, whereas the peak at 1062 cm^−1^ is attributed to C–O stretching [15, 36].

Figures 1(b) and 1(c) showed characteristic peaks of C934 and the physical mixture of CTX and C934 in 1 : 1 ratio. FTIR spectrum of the mixture revealed that there is neither appearance of new peaks nor disappearance of existing peaks, which indicated that there is no interaction between the drug and the gelling agent used. Characteristic peaks of the drug groups were identified in all the spectra of prepared physical mixtures of the drug and different polymers used as gelling agents (not included). This study revealed that the drug was compatible with each of the selected gelling agents and indicated the suitability of the selected polymers for preparation of topical CTX gel formulation.

### 3.2. Characterization of the Prepared CTX Hydrogels

Results of the visual inspection of the prepared CTX hydrogels are presented in Table 2. Most of the prepared hydrogels were yellowish in color, either opaque or translucent or transparent. All the prepared hydrogels showed good homogeneity with absence of lumps and syneresis. No signs of grittiness. The hydrogels had smooth homogenous texture and glossy appearance except F10 which showed clumps.

pH values of the prepared formulas were in the range of 5-6 which is considered acceptable to avoid skin irritation upon application [37]. PEC-based gel was an exception as pH was about 4. Additionally, there was no significant change in pH values as a function of time for all formulations. Results are illustrated in Table 2.

The data presented in Table 2 showed that the percentage drug content of prepared hydrogel formulations ranged from 95 to 98.9% which are within the official limits (100 ± 5%) [38]. This indicated that the drug was uniformly distributed throughout the gel. Therefore, the method used in the present study seems to be reproducible for the preparation of CTX hydrogels.

**Figure 2:**
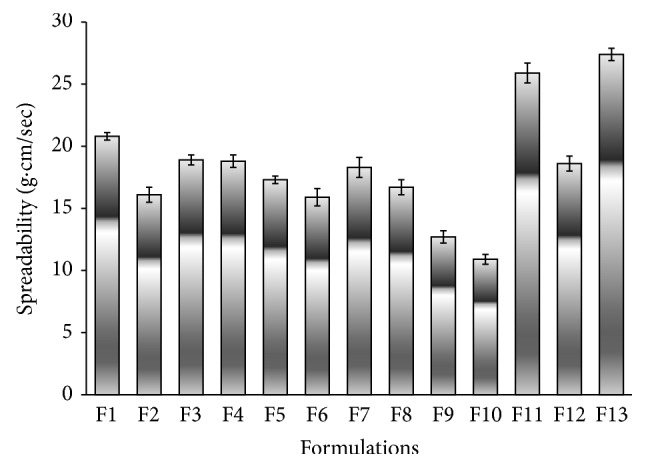
Spreading coefficient of different hydrogel formulations F1–F13.

Good spreadability is one of the criteria for gel to meet ideal qualities. It is the term expressed to denote the extent of area to which gel readily spreads on application. Therapeutic efficacy of a gel formulation also depends on its spreading value [22]. Additionally, spreadability is very important as it shows the behavior of the gel when it comes out from the tube [39]. Spreading coefficient of different hydrogels is shown in Figure 2. The determined spreadability values indicated that the polymers used provided hydrogels which can spread by shearing force of low magnitude. It was observed that spreadability of CTX hydrogels decreased by increasing the polymer concentration and the values were in the range of 10.9 to 27.4 gm·cm/sec. Maximum spreadability was observed for hydrogels containing C934 (2% w/w), followed by that containing HPMC 4000 (3% w/w) and that formulated with 2% w/w XG. The lowest spreadability was for Na CMC (4 and 7%).

It can be concluded that the prepared hydrogels fulfilled the requirement of gel-based formulations for dermatological use which should have several favorable properties such as greaseless and ease of spreadability [3, 17].

**Figure 3:**
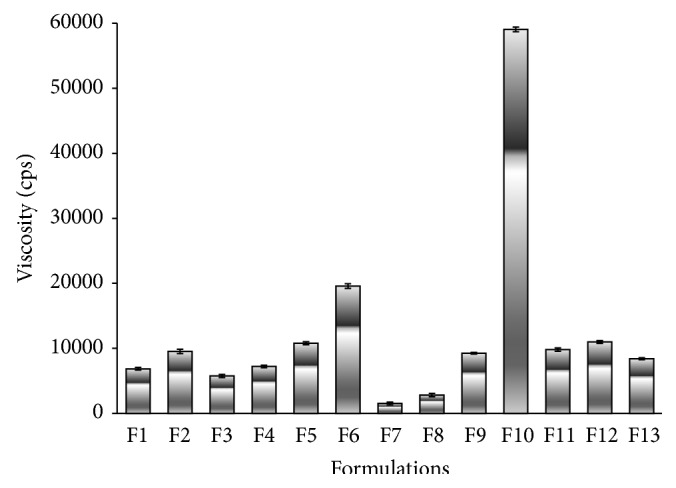
Viscosity in centipoises of different hydrogel formulations F1–F13.

It was reported that viscosity is an important physical property of topical formulations, which affects rate of drug release [20]. It was found that viscosity of the prepared hydrogels ranged from 1522 to 59042 centipoises. The viscosity increased upon increasing the polymer concentration (Figure 3). The lowest viscosity was observed with hydrogels containing PEC while hydrogels containing Na CMC showed the highest viscosity. Viscosity of hydrogels prepared with GG was changed with time. Generally, it was observed in all hydrogels that when the rate of shear is increased the viscosity decreases which indicated that the formulation is shear thinning pseudoplastic in nature.

### 3.3. *In Vitro* Release Study

**Figure 4:**
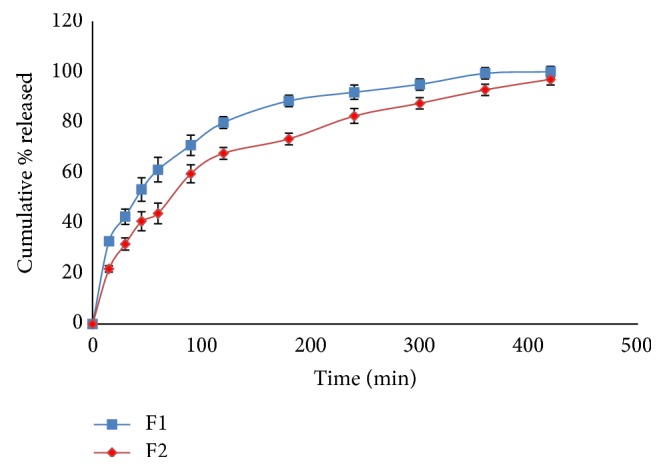
Drug release profiles of hydrogel formulations F1 and F2.

**Figure 5 fig5:**
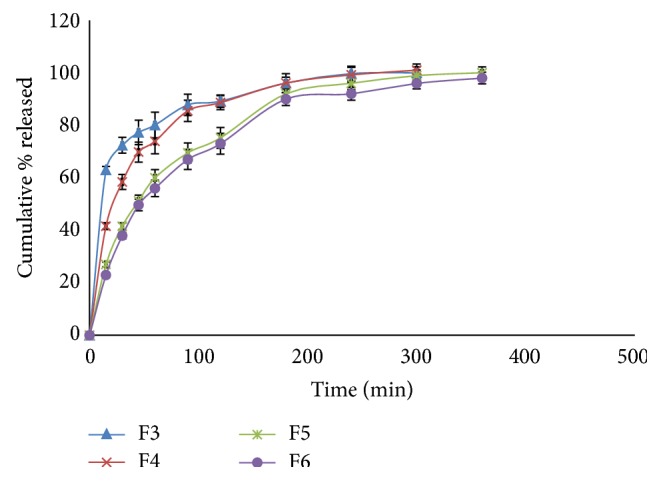
Drug release profiles of hydrogel formulations F3–F6.

**Figure 6 fig6:**
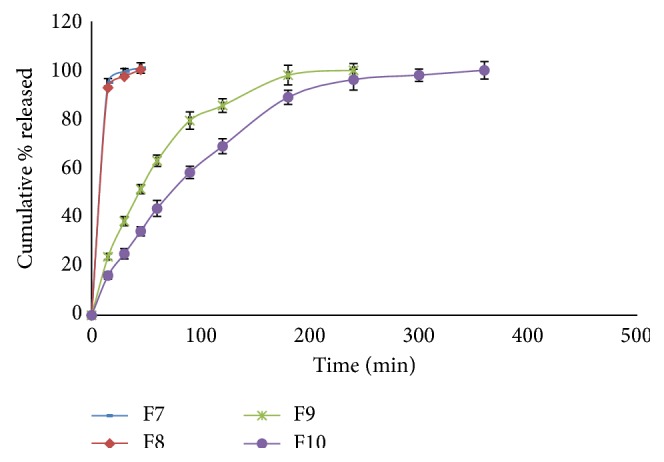
Drug release profiles of hydrogel formulations F7–F10.

**Figure 7:**
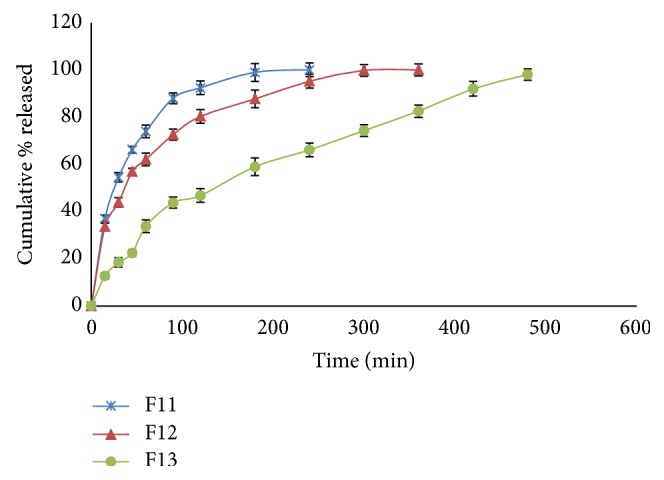
Drug release profiles of hydrogel formulations F11–F13.

The release profiles of CTX from different hydrogel formulations are shown in Figures 4 [Fig fig5] [Fig fig6]–7. It is obvious that the release of CTX from hydrogels was varied according to concentration of the polymer and viscosity of the hydrogels. Generally, an increase in the vehicle viscosity would cause a more rigid structure with a consequent decrease in the rate of drug release [16]. Hydrogels F7 that exhibited the lowest viscosity (1522 centipoises) released its drug content within 30 min, while hydrogels F10 which had the highest viscosity (59042 centipoises) showed 100% drug release within 6 h.

The release of the drug from the prepared hydrogels was ranked in case of hydrogels containing XG in the order F1 > F2 and the release was more than 95% after 6 h. In case of GG-based hydrogels, the order of release was F3 > F4 > F5 > F6, depending on the amount of CTX released after 5 h. This result can be explained by the increase in polymer concentration from 2 to 5% w/w which resulted in an increase in the hydrogel viscosity from 5766 to 19582 centipoises. Hydrogel F3 released about 80% of its drug content within one hour but hydrogel F6 released about 56% of its drug content after the same time. It is clear that as the viscosity increased by approximately 4-fold, a decrease in the drug release by approximately 1.5-fold was observed.

Same results were obtained for the other prepared hydrogels. Drug release from PEC-based hydrogels, namely, F7 and F8, was very fast as more than 95% of the drug content was released within 45 min. Hydrogel F7 showed faster and higher drug release than formulation F8. In case of hydrogels containing Na CMC, the release was ranked in the order F9 > F10 and complete drug release was attained within 4 and 5 h, respectively. Drug release behavior of hydrogels containing HPMC 4000 was in the order of F11 > F12 and complete drug release was achieved after 4 and 6 h, respectively, whereas the drug release from hydrogel F13 containing C934 was slow and sustained for 8 h.

It was also observed that the drug release did not depend only on hydrogels' viscosity but it was also influenced by the type and network structure of each polymer used. For example, the viscosities of F2 (XG-based hydrogel) and F11 (HPMC-based hydrogel) are nearly in the same range (9537 and 9817 centipoises) but F2 released about 93% of its drug content within 6 h, whereas F11 released its drug content completely after 4 h. On the other hand, hydrogels F5 (GG-based hydrogel) and F12 (HPMC-based hydrogel) with a viscosity of 10788 and 10981 centipoises, respectively, both released about 95% of their drug content after 4 h. Increasing viscosity to 59042 centipoises as in case of hydrogel F10 (Na CMC-based formulation) released the same amount of the drug at the same time (4 h).

Among all hydrogel formulations, C934 (F13) showed superior sustained drug release followed by HPMC 4000 (F11) and XG (F1). C934 is a hydrophilic polyacrylic acid polymer and its carboxyl groups become highly ionized after neutralization with TEA, forming a gel due to electrostatic repulsion among charged polymer chains. Increasing pH of the prepared hydrogel to be suitable for skin application resulted in uncoiling of the polymer chains due to ionization of its carboxyl groups and subsequently forming a rigid gel [40]. The colligative results revealed that Carbopol was a good gelling agent as compared to other agents for hydrogels' preparation. These results were consistent with previously reported work [41–43].

Results of* in vitro* release study verified that the most important factors influencing CTX release from the prepared hydrogels are polymer type and concentration. Polymer type proved to have a remarkable and predominant influence on the drug release from the prepared hydrogels followed by the polymer concentration. Many studies showed that drug release was decreased with an increase in gelling agent concentration. As the polymer concentration increases, viscosity increases [3, 20, 39].

The progressive decrease in the amount of drug released from hydrogels, as the release test proceeded, was attributed to gradual increase in concentration of the eroded polymer which increased the viscosity of the system. Consequently, the diffusion of the drug through the membrane decreased.

#### 3.3.1. Kinetic Modeling of Drug Release

Different mathematical models were used to describe the kinetics of CTX hydrogels. The values of the release exponent (*n*) and the regression values of zero-order, first-order, and Higuchi release models for different formulations are represented in Table 3.

The Higuchi kinetic plots were found to be fairly linear as indicated by their highest regression values. The correlation coefficients values (*R* ^2^) were in the range of 0.843–0.995 for all hydrogel formulations. In general, it was observed that the mechanism of drug release of all formulations was either anomalous diffusion or non-Fickian super case II.

The release exponents (*n*) for optimized formulations F1, F11, and F13 were found to be 1.330, 1.069, and 0.860, respectively. The diffusion exponents *n* for F1 and F11 were more than 1, which indicates a non-Fickian super case II. On the other hand, formulation F13 showed a diffusion exponent of 0.860 (0.5 < *n* < 1), indicating anomalous diffusion coupled with erosion.

### 3.4. Stability Study for the Formulated CTX Hydrogels

CTX hydrogels (F1, F11, and F13) which showed promising results as sustained release formulations were subjected to stability studies at refrigerator and ambient room conditions for 3 months. After storage for 3 months, hydrogels did not show any change in color, odor, pH, drug content, and rheological properties. Additionally, no phase separation occurred. This indicated that the drug was stable in gels even after 3 months of short term storage and the gel formulations were physically and chemically stable.

It is clear from above discussion that F1, F11, and F13 are the best formulations among all the prepared formulations; therefore, they were subjected for further investigation for microbiological activities.

### 3.5. Microbiological Studies

SSTIs are considered as one of the serious problems worldwide, especially due to bacterial resistance. Shortage in marketed topical dosage forms containing different groups of antibiotics aroused the need of oral and parenteral applications with their accompanied side effects.

Cefotaxime (CTX) sodium as one of cephalosporin broad spectrum antibiotics has a high safety margin and was reported to be effective against different multidrug resistant pathogen as by the FDA.

In the present study, effect of the prepared CTX topical gel formulations was investigated through different microbiological techniques.

#### 3.5.1. Evaluation of Bacterial Susceptibility to CTX Gel Formulations

*(1) Determination of Minimal Inhibitory Concentration (MIC)*. Results of broth microdilution method with different CTX formulations were represented in Table 4. The formulas F1, F11, and F13 which showed considerable good spreadability and release characteristics were selected further to determine their MIC against some different bacteria isolated from wound and SSTIs. A standard strain of each of the tested Gram negative bacteria was studied as well for the sake of fair judgment to the selected formulas' effect. Furthermore, standard* S. aureus* was tested with one MRSA strain for a better indication of the antimicrobial coverage of CTX gel formulas. CTX pure drug was tested along with the gel formulas as a control (Table 4). Results revealed that the MIC values of the standard strains obtained were consistent with the interpreted CLSI 2011 values. F13 showed comparable MICs to the control drug and showed better efficacy than F1 and F11. The MICs against standard strains of* E. coli* and* P. aeruginosa* were from 0.06–2 to 31.25–62.5 *μ*g/mL, respectively, while it was from 1–>2 and from 62.5–250 *μ*g/mL against clinical isolates of the same strains, respectively.* S. aureus* showed much lower MICs of all formulations tested when compared to the isolated MRSA where the range was from 0.25–1 to 7.8–31.25, respectively. Analysis of the results indicated that isolated* P. aeruginosa* gave the highest MIC values but it is still considered as having an intermediate sensitivity to CTX as previously reported [44, 45]. These obtained minimum inhibitory values against the bacterial isolates were consistent with previously reported results [46, 47]. The gel base did not show any antibacterial activity.

**Figure 8:**
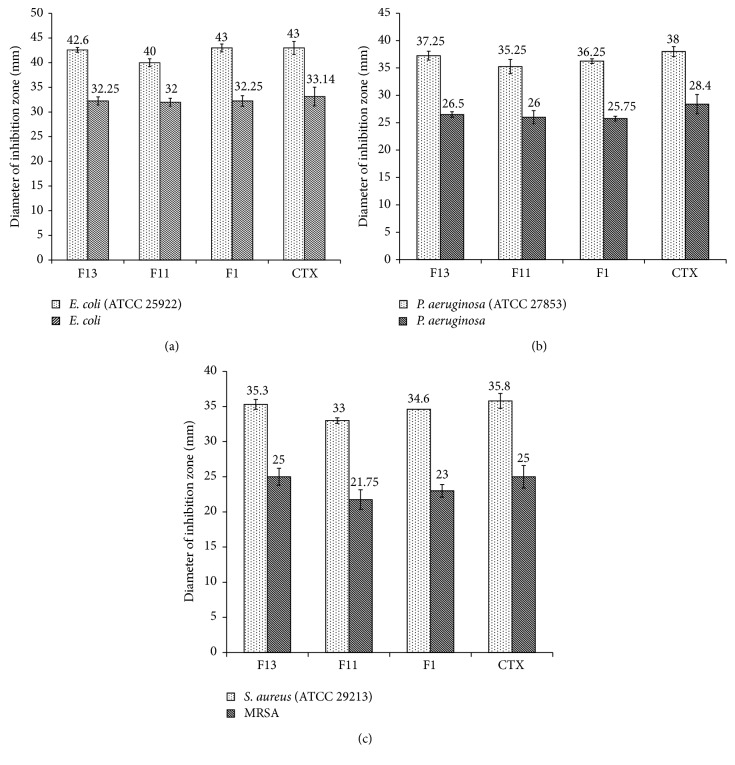
Agar well diffusion assay for antibacterial activities of different CTX gel formulations against selected pathogens.

*(2) Agar Well Diffusion Assay*. Agar well diffusion method was used for screening the antimicrobial potential of CTX gel formulations and the results were shown in Figure 8. Effect of the three chosen formulas on the selected bacterial strains was represented as the mean of three readings (mm ± SD). Along with the formulas, control pure drug was tested as well for better comparison. Figure 8 clearly demonstrated that there was no significant difference in zone of inhibition between F1, F11, and F13. Diameter of inhibition zones shown by formulation F13 was similar to that of crude drug against any of the strains tested which is in agreement with the fact that incorporation of the drug into gel base does not decrease its antibacterial activity [48]. Additionally, this can be attributed to the great spreadability characteristic of F13 gel which affects therapeutic efficiency of the drug.

Tested formulas showed significant results (*P* < 0.05) against Gram negative standard strains tested (Figures 8(a) and 8(b)) compared to isolated one (Ca. 10 mm difference).

Although the tested formulations demonstrated better effect against* E. coli* isolate as compared with* P. aeruginosa* isolate, the obtained* P. aeruginosa* results were in the acceptable susceptibility values to CTX as revised in CLSI [49].

Figure 8(c) showed the effect of three formulations against Gram positive* S. aureus*. Two strains were tested,* S. aureus* and MRSA. Against* S. aureus*, the zones of inhibition were significantly better than MRSA, giving more than 10 mm difference. F11 showed the least activity against both* S. aureus* and MRSA, though it was insignificant (Figure 8(c)).

Generally,* E. coli* standards were the most affected strains by gel formulations as compared with either* P. aeruginosa* or* S. aureus*.

Same results were found by Anacona and Da Silva as they found that the zone of inhibition of CTX on* E. coli* was 50 mm while it was 26 and 30 for* P. aeruginosa* and* S. aureus*, respectively [50]. On the other hand, using bacterial isolates, Manikandan and Amsath reported that the percentage of resistance of* E. coli* to CTX was 87.5 while it was only 16.7 for* P. aeruginosa* and even 0% for* S. aureus* [46]. However, it was previously mentioned that CTX retained high levels of antipseudomonal activity compared to other antibiotics used [51]. Although MRSA is found to be resistant to many routinely used antibiotics, in this study it showed intermediate susceptibility to CTX. This result was in accordance with Lakshmi et al. who found that about 40% of MRSA isolates used were susceptible to CTX [32]. Furthermore, it was recently reported that out of 58 MRSA strains 16 were sensitive to CTX [52]. Also, Wareg et al. calculated the % susceptibility of outpatient MRSA to be 70% intermediate while only 20% was resistant and 10% was sensitive [53].

#### 3.5.2. Effect of CTX Gel Formulations on Bacterial Pathogens Survival

**Figure 9:**
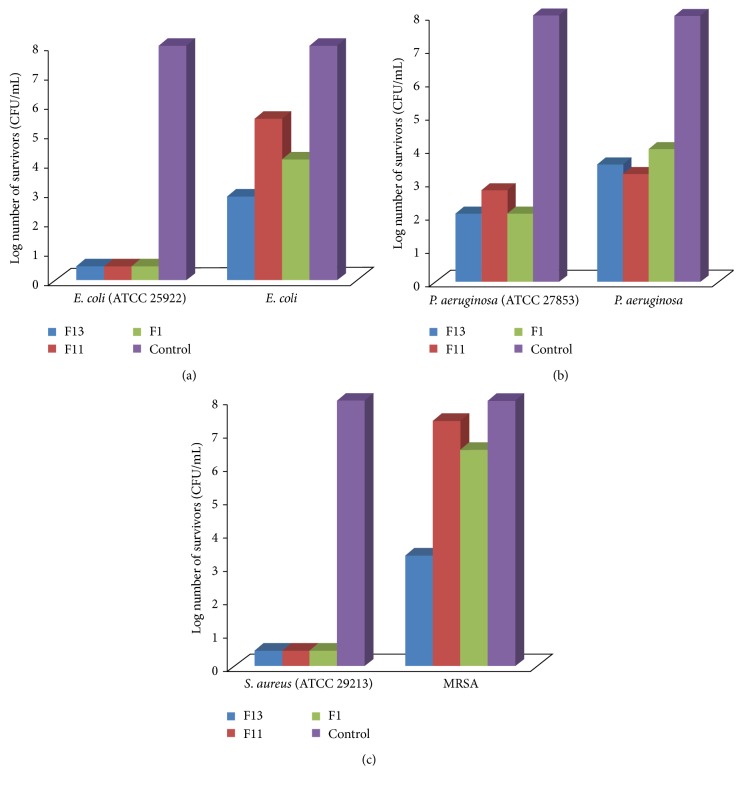
Killing pattern of different CTX gel formulations against* E. coli*,* P. aeruginosa*, and* S. aureus*.

The action of the three topical gel formulations containing 1% CTX was evaluated against the previously selected pathogens. Total killing was noticed after 24 h by using any of the selected formulas against* S. aureus* (Figure 9(c)) and* E. coli* (ATCC 25922) (Figure 9(a)). Against standard* P. aeruginosa* there was a decrease in number of survivors of more than 5-log reduction compared to the control by application of F11 and even more than 6-log reduction caused by other two formulas (F1 and F13) (Figure 9(b)).

Against isolates used, different results were obtained. An inhibition of about 4-log difference than control was obtained by F1 against* P. aeruginosa* and* E. coli*. Only 2-log reduction was obtained by F11 against* E. coli* but the difference was more than 5-log reduction against* P. aeruginosa*.

C934-CTX gel formulation (F13) showed the most bactericidal effect against both* E. coli* and* P. aeruginosa*, giving more than 5-log reduction difference compared to control.

On the other side, MRSA was almost not greatly affected by application of F11 and F1 since the difference in log number of survivors was less than 2 log. However, upon application of F13, the reduction in CFU was significant compared to control reading (>5 log).

Observed variation in formulas' effect on the isolates may be related to the difference in nature of the three polymers used in the formulas concerning their chemical structure and viscosity. Furthermore, good spreadability of F13 formula compared to F1 and F11 can be a probable cause for better killing activity since spreadability directly affects therapeutic efficacy of the drug. The present results were consistent with previously reported work done by Dua et al. [54]. They found that Carbopol (C934) gel-based formulations exhibited better antibacterial activity against* P. aeruginosa*,* S. aureus*, and* E. coli* in comparison to other dermatological base formulations, indicating better activity of the drug. Moreover, it was reported that C934 based gel proved to be the polymer of choice since it showed the highest antimicrobial activity when compared to other gel-based formulations [22, 39]. For the above reasons, F13 was the formula of choice to investigate its efficacy in wound treatment.

#### 3.5.3. *In Vivo* Study for Evaluation of C934-CTX Efficacy in Wound Treatment

*In vivo* studies were performed to further prove the efficacy of F13 on eradication of Gram negative or Gram positive bacteria from an infected rat wound model.* P. aeruginosa* and MRSA were chosen on the basis that they were found to be the most prevalent pathogens isolated from wound infection [46].

**Figure 10:**
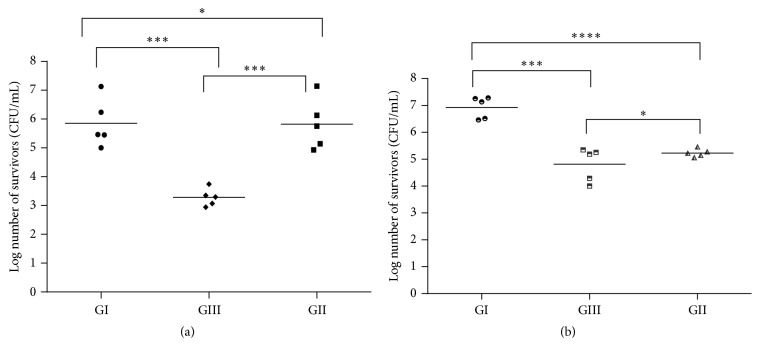
Killing curve for the prepared topical gel F13 (GIII) against (a)* P. aeruginosa* and (b) MRSA in comparison with control group (GI) and Fucidin treated group (GII) ^*∗*^ *P* > 0.05, ^*∗∗∗*^ *P* < 0.002, and ^*∗∗∗∗*^ *P* < 0.001.

*(1) Killing Curve Using Skin Homogenate*. Figures 10(a) and 10(b) show the recovery of* P. aeruginosa* and MRSA strain, respectively, after 24 h of inoculation with approximately 1 × 10^8^ CFU/mL bacteria followed by single treatment with either F13 (GIII) or marketed fusidic acid cream (Fucidin) (GII). Five animals were left untreated in each group for the sake of proper comparison (GI). The number of CFU/mL in tissue homogenate was determined by surface viable count technique. Regarding the recovery of* P. aeruginosa* strain (Figure 10(a)), there were significant differences (*P* < 0.002) between F13 (GIII) treated tissue and both the control tissue (GI) and Fucidin treated ones (GII) (>3-log reduction). On the other side, there was almost no noticeable difference between the numbers of* P. aeruginosa* cells recovered from control tissue and the ones recovered after Fucidin treatment. Regarding the MRSA strain infected animal tissue, the results were different. There was a significant difference of more than 2-log reduction between the control group and both F13 treated and Fucidin treated tissues (*P* < 0.002 and *P* < 0.001, resp.). Roche et al. reported that there was a >3-log reduction upon application of mupirocin and bacitracin topical antibiotics after 4 h of MRSA inoculation. These previously* in vivo* count results are consistent with the observed data from the killing diagram (Figure 10) [34].

**Figure 11:**
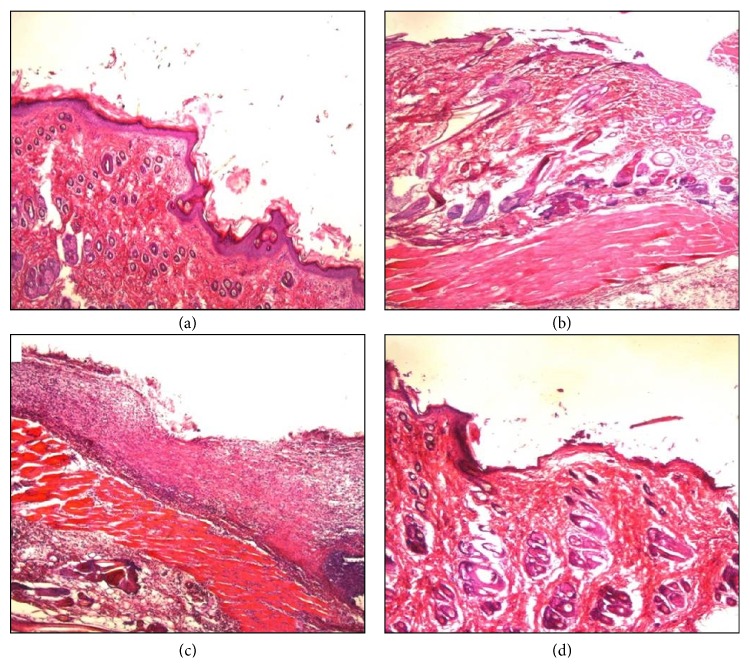
Microscopic appearance of skin tissues sections after H&E staining, 500x. (a) Untreated skin tissues of rat after 24 h microbial inoculation with MRSA isolates. (b) Skin tissue treated with F13 gel formulation after 24 h microbial inoculation with MRSA isolates. (c) Untreated skin tissues of rat after 24 h microbial inoculation with* P. aeruginosa* isolates. (d) Skin tissue treated with F13 gel formulation after 24 h microbial inoculation with* P. aeruginosa* isolates.

*(2) Histological Study*. To confirm our previous findings, histopathological study was performed by taking 1 cm^2^ of animal tissue after 24 h of microbial inoculation. This was done by comparing the thickness of microbial bioburden between the treated and untreated tissues. Moreover, eradication of the infection by F13 gel formulation was assessed. The tissues were stained with Hematoxylin and Eosin. Results were shown in Figures 11(a) and 11(b) for MRSA isolate and Figures 11(c) and 11(d) for* P. aeruginosa* isolate.

Untreated tissues (Figures 11(a) and 11(c)) showed a thick film of microbial colonization and partial skin structure damage after 24 h of incubation. However, after single treatment with F13 gel formulation, a noticeable reduction in microbial bioburden occurred in case of both Gram positive isolate (Figure 11(b)) and Gram negative isolate (Figure 11(d)). This could be attributed to the extended drug release of F13 which assisted drug penetration to skin tissue and increased the duration of action of the drug and its bioavailability. This explanation was in consistence with previously reported studies in which C934 served as a convenient polymer for topical gel formulations providing extended drug release for a long period of time [42, 43].

## 4. Conclusion

SSTIs are considered a problem in the field of medicine for a long time. Third-generation antibiotic, Cefotaxime (CTX), proved to be very effective against most strains of bacterial pathogens.

In the present investigation, CTX was successfully incorporated into different gel formulations. Among all gel formulations, CTX gel (F13) prepared from C934 (2%) as gel reservoir proved to be the formula of choice, showing good characteristics and controlling the drug release for long period of time. Furthermore, it showed promising antibacterial activity* in vitro* against* P. aeruginosa*,* E. coli*,* S. aureus*, and MRSA. Moreover, it was highly efficient in eradicating* P. aeruginosa* and MRSA induced dermal infections.

CTX gel formulation F13 could be very promising and innovative topical alternative for treatment of skin infections caused by Cefotaxime-susceptible bacteria and play a vital role in drug efficiency. These findings may open new avenues for the treatment of dermal infections by local application of tailored antibiotic gel. However, further preclinical and clinical studies are recommended to support its efficiency claims in humans.

## Figures and Tables

**Table 1:** Composition of CTX topical hydrogels (% w/w).

Ingredients	F1	F2	F3	F4	F5	F6	F7	F8	F9	F10	F11	F12	F13
CTX	1	1	1	1	1	1	1	1	1	1	1	—	—
XG	2	3	—	—	—	—	—	—	—	—	—	—	—
GG	—	—	2	3	4	5	—	—	—	—	—	—	—
PEC	—	—	—	—	—	—	6	7	—	—	—	—	—
Na CMC	—	—	—	—	—	—	—	—	4	7	—	—	—
HPMC	—	—	—	—	—	—	—	—	—	—	3	4	—
C934	—	—	—	—	—	—	—	—	—	—	—	—	2
PG	5	5	5	5	5	5	5	5	5	5	5	5	5
Sodium benzoate	0.25	0.25	0.25	0.25	0.25	0.25	0.25	0.25	0.25	0.25	0.25	0.25	0.25
Purified water to	100	100	100	100	100	100	100	100	100	100	100	100	100

**Table 2:** Physical properties of CTX topical gel formulations (F1–F13).

Formulations	Color	Homogeneity	Grittiness	pH	% drug content
F1	Opaque, yellowish	+++	—	6.6	96 ± 0.3
F2	Opaque, yellowish	+++	—	6.5	98.8 ± 0.1
F3	Translucent yellowish	+++	—	5.9	95.4 ± 0.2
F4	Translucent yellowish	+++	—	5.6	98.9 ± 0.3
F5	Translucent yellowish	+++	—	5.7	97.5 ± 0.4
F6	Translucent yellowish	+++	—	5.6	97.8 ± 0.1
F7	Opaque, buff	+++	—	4.0	98 ± 0.2
F8	Opaque, buff	+++	—	4.0	98.3 ± 0.1
F9	Shiny, transparent, yellowish	+++	—	6.2	97.5 ± 0.2
F10	Shiny, transparent, yellowish	Clumpy	—	6.3	95 ± 0.1
F11	Transparent	+++	—	5.7	98 ± 0.3
F12	Transparent	+++	—	5.9	97.8 ± 0.4
F13	Transparent	+++	—	6.3	96 ± 0.2

++: good; +++: very good; —: no grittiness.

**Table 3:** Kinetic study of the *in vitro* release data of prepared CTX hydrogels.

Formulation code	Zero-order	First-order	Higuchi model	Korsmeyer-Peppas model (*n*)
	Correlation coefficient (*R* ^2^)			
F1	0.848	0.652	0.976	1.330
F2	0.913	0.663	0.990	0.834
F3	0.689	0.644	0.843	2.510
F4	0.785	0.603	0.922	1.597
F5	0.875	0.647	0.975	1.256
F6	0.881	0.648	0.978	1.330
F7	0.805	0.241	0.936	8.730
F8	0.812	0.179	0.940	11.15
F9	0.896	0.589	0.980	0.629
F10	0.927	0.661	0.993	0.844
F11	0.820	0.579	0.943	1.069
F12	0.857	0.655	0.967	1.421
F13	0.969	0.693	0.995	0.860

**Table 4:** MIC (*µ*g/mL) of different CTX gel formulations against Gram positive and Gram negative standard and clinical isolates.

Bacterial Strains	CTX	F13	F11	F1
*E. coli* (ATCC 25922)	0.06	0.06	1	2
*E. coli*	1	1	>2	>2
*P. aeruginosa* (ATCC 27853)	31.25	31.25	62.5	31.25
*P. aeruginosa*	62.5	125	250	62.5
*S. aureus* (ATCC 29213)	0.25	0.25	0.5	1
MRSA	7.8	15.6	31.25	31.25
